# Medrysone promotes corneal injury repair by promoting M2-like polarization of macrophages

**DOI:** 10.1186/s12886-023-03234-3

**Published:** 2023-12-11

**Authors:** Yaqin Zhu, Xiaohong Jin, Ning Fu, Jiuke Li

**Affiliations:** 1 Hangzhou Aier Eye Hospital, Hangzhou, 310000 Zhejiang China; 2 Sir Run Run Shaw Hospital Hangzhou, Hangzhou, 310000 China

**Keywords:** Corneal Injury, Medrysone, M2 polarization

## Abstract

**Background:**

Accumulated evidence suggests that M2-like polarized macrophages plays an important role in reducing inflammation, promoting and accelerating wound healing process and tissue repair. Thus, M2-like TAMs (Tumour-associated macrophages) was an appealing target for therapy intervention.

**Method:**

Flow cytometry and RT-PCR assay were used to detect the polarization of macrophages induced by Medrysone, and the rat corneal mechanical injury model was established to evaluate the efficacy of Medrysone in cornel repair.

**Results:**

Here we found that Medrysone enhanced IL-4 induced M2 polarization of macrophages, as illustrated by increased expression of CD206, up-regulation of M2 marker mRNAs. Medrysone promoted VEGF and CCL2 secretion in IL-4 induced M2-like polarization. IL-4 triggered STAT6 activation was further enhanced by Medrysone and silencing of STAT6 partially abrogated the stimulatory effect of Medrysone. Medrysone improved migration-promoting feature of M2-like macrophages, as indicated by increased migration of endothelial cells. Further, Medrysone promoted corneal injury repair by inducing M2 polarization of macrophages in vivo.

**Conclusion:**

Our study suggest that Medrysone promotes corneal injury repair by inducing the M2 polarization of macrophages, providing a theoretical basis for the application of Medrysone in the treatment of corneal injury.

**Supplementary Information:**

The online version contains supplementary material available at 10.1186/s12886-023-03234-3.

## Background

The cornea is located at the forefront of the eyeball. The corneal is one of the most important parts of the surface barrier and refractive imaging system of the eyes, and corneal damage occurs frequently and severely affects the vision [1]. Long-term corneal injury may cause corneal scarring, opacity, and even blindness [2, 3]. Corneal injury repair is a complex biological process that mainly involves three processes: the inflammatory response, tissue proliferation, and wound shrinkage and scarring [4]. During corneal repair, inflammation is frequently activated to prevent infection and promote tissue repair, while excessive inflammation can lead to impaired corneal healing. Therefore, promoting rapid repair of the injury and avoiding excessive inflammatory response are important research topics in clinical ophthalmic treatment [5].

Acute inflammation often occurs during corneal injury repair. When the cornea is damaged or infected, macrophages migrate from the limbal vascular network to the limbus or central corneal region in response to chemokines secreted by keratocytes, and protect against external invasion [6]. During the acute phase of inflammation, classic M1-like macrophages are activated to induce inflammation and release proinflammatory mediators, such as cytokines, chemokines, and reactive oxygen and nitrogen intermediates, which help pathogens kill and exert anti-inflammatory effects [7]. However, these responses must be controlled to prevent overreactions that damage the host. At this time, M2-like macrophages are activated to avoid serious immune pathology such as anti-inflammatory cytokine mediators, cytokines and chemokines that are regulated by M1-like macrophages, thereby reducing inflammation, promoting and accelerating wound healing process and tissue repair [8, 9]. This fine-tuned balance between M1 and M2 helps host cells cope with stress, inflammation, regression and repair.

Corticosteroids are widely used in the treatment of various keratitis [10]. The main mechanism of corticosteroids executes an anti-inflammatory effect to reduce haze and scarring, including the treatment of post-refractive surgery and herpes simplex stromal keratitis [11]. In this study, we found that the corticosteroids drug Medrysone can promote corneal injury repair by inducing the M2 polarization of macrophages. M2-like macrophages promoted the secretion of VEGF (Vascular Endothelial Growth Factor) and the migration of vascular endothelial cells, thereby induced the cornea wound healing repair. This study provides a theoretical basis for the application of Medrysone in the treatment of corneal injury.

## Materials and methods

### Antibodies and reagents

Medrysone (T0977, TargetMol), IL-4 (51,084-MNAE, Sino Biological), APC anti-mouse CD206 (#141,708, Biolegend), PE anti-mouse CD206 (#141,706, Biolegend), FITC anti-mouse F4/80 (#123,108, Biolegend), M-CSF (#315–02-50, PeproTech), GAPDH (db106, Diagbio), STAT6 (#9362, Cell Signaling Technology), p-STAT6 (#9361, Cell Signaling Technology).

### Cell culture

RAW264.7 cells were obtained from the Cell Bank of the China Science Academy (Shanghai, China) and C166 cells were purchased from ATCC. RAW264.7 cells and C166 cells were cultured in DMEM medium respectively containing 10% fetal bovine serum in a 5% CO_2_ humidified incubator at 37℃.

### Bone marrow-derived macrophages (BMDMs) isolation and differentiation

C57BL/6 (4–6 weeks old) male mice purchased from Beijing Vital River Laboratory Animal Technology Co., Ltd (Beijing, China) were sacrificed for obtaining bone marrow. Mice were euthanized by cervical dislocation, and subsequently collected bone marrow cells from the tibia of mice. The isolated bone marrow cells were administrated with macrophage colony-stimulating factor (M-CSF, 50 ng/mL) and cultured in DMEM with 10% fetal bovine serum for five days to get BMDMs. Generally, 2–3 × 10^7^ BMDMs can be obtained from per mouse. This experiment was reviewed and approved by the Institutional Animal Care and Use Committee (IACUC) on the Ethics of Animal Experiments of Zhejiang University (Permit number: 19NGJZ050Mou).

### Animal study

Six- to eight-week-old male rats were purchased for establishing corneal injury model. Rat corneal mechanical injury was established by using ophthalmic surgical instruments. After the operation, the rat corneas were stained with sodium fluorescein (0.5% in PBS), and recorded by direct focal illumination using a slit lamp. Then, Rats were randomly divided into 2 groups, and treated with Medrysone (5 μM) every 6 h, and after 48 h, recorded the corneal damage by fluorescein sodium staining again. The corneal tissue was collected, and the expression of Mrc1, Fizz1, Arg1 and CD11b mRNA level was detected by RT-PCR assay (real-time PCR assay). This experiment was reviewed and approved by the Institutional Animal Care and Use Committee (IACUC) on the Ethics of Animal Experiments of Zhejiang University (Permit number: 19NGJZ050Mou).

### Corneal injury model [12]

For establishing corneal injury model, rats were anesthetized by using Isoflurane. Rat corneal injury was constructed using a #11 surgical blade (#69,901,721, shanghai jinzhong metal products co., ltd). Briefly, laboratory technician used the tip of surgical blade to cause a linear injury on the rat cornea, and repeated this operation 10 times.

### Flow cytometry

RAW264.7 cells or BMDMs were collected and blocked with 3% BSA for 45 min at 4℃, then were washed and incubated with PE-CD206 and FITC-F4/80 antibodies according to the manufacturer’s instructions. About 1 × 10^4^ cells of each sample were analyzed by using the BD FACS Verse cytometer (Becton Dickinson, SanJose, CA).

### Real-time PCR assay

The total RNA extraction from RAW264.7 cells and BMDMs were obtained by using Trizol (Invitrogen, CA, USA), and using TransScript One-Step gDNA Removal and cDNA Synthesis Super Mix (TransGen Biotech, Beijing, China) kit to synthesize cDNA. The real-time PCR assay was conducted by SYBR Green Master Mix (Bio-Rad Laboratories, CA, USA). GAPDH mRNA was used as control to evaluate relative the level of target genes.

### Conditioned medium preparation

RAW264.7 was cultured in DMEM with 10% FBS and administrated Medrysone (5.0 μM), IL4 (10 ng/mL), or both of them for 24 h. Then discard the supernatants and change into fresh serum-free medium to culture for another 24 h, then collected the supernatants of these cells as conditioned medium (CM).

### Trans-well assay

The migration assay was performed using a Trans-well Boyden Chamber (Costar, Bethesda, MD, USA). C166 cells (3 × 10^4^ /well) in 200 μL CM were seeded into the upper chamber, and the same CM (600 μL) was placed in the lower chamber to incubated at 37 °C for 24 h. Then, cells on the upper chamber were fixed with cold 75% ethyl alcohol for 45 min at room temperature, followed by staining with 0.1% crystal violet. The non-migrant cells on the upside of the upper chamber were removed. The migrant cells on the bottom of the upper chamber were photographed by a microscope. Randomly select four fields of each specimen were photographed by a microscope for analyzing data by Image Pro Plus 6.0.

### Wound healing assay

Murine vascular endothelial C166 were seeded in 24-well plates and cultured until 70–80% confluent. Use a pipette tip to make a straight scratch and format an artificial wound. Cells were incubated with conditioned medium for 24 h. Randomly select four fields of each specimen (10 × magnification) were photographed to analyze the migration of cells, by using a LEICA DMI 4000B microscope with Leica Application Suite software.

### Cell transfection

The siRNA sequence was duplexes produced by Genepharma, Co. (Shanghai, China). The sequences of siRNAs used were as follows, si-STAT6: AGCAGGAAGAACTCAAGTTTA. The transfection was performed using siRNA and jet Prime according to the manufacturer’s recommendations.

### Western blotting

Western blotting was conducted as previously described. Primary antibodies against STAT6, GAPDH and p-STAT6 were used at 1:1000 in dilution.

### Statistical analyses

All experiments were analyzed by three independent experiments. Data were analyzed by one-way ANOVA with Dunnett’s post hoc test and Students’ t test. Differences were statistically significant when the *p*-value was less than 0.05.

## Results

### Medrysone promoted M2 polarization of Macrophages induced by IL-4

**Fig. 1:**
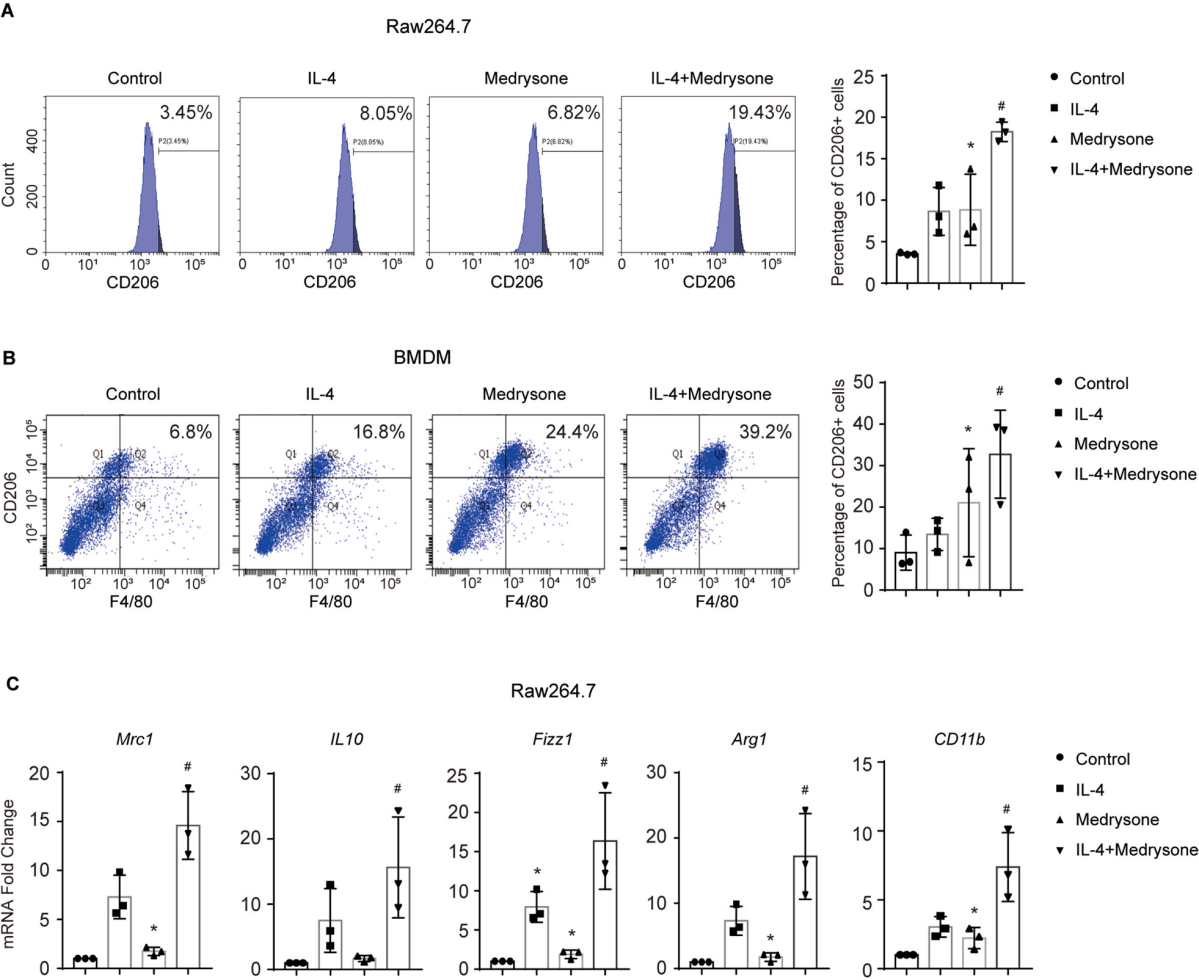
Medrysone induced M2 polarization of Macrophages. **A** and **B** Medrysone induced the percentage of CD206-positive cells. Raw264.7 and BMDMs were treated with or without Medrysone (5 μM) and IL-4 (10 ng/mL) for 24 h and the expression of CD206 was detected by flow cytometry. **C** Medrysone promoted the M2-like-associated genes expression. The mRNA level of *Mrc1, IL10, Fizz1* and* Arg1* was detected by quantitative real-time PCR assay after the treatment of Medrysone (5 μM) and IL-4 (10 ng/mL) for 24 h. The data are presented as the mean ± SD of triplicate experiments. Statistical significance was determined by one-way ANOVA with Dunnett’s post hoc test. *, #, p < 0.05

According to previous reports, M2-like macrophages played an important role in wound healing and tissue repair [13]. Thus, we investigated the effect of the corticosteroid Medrysone on the M2 polarization of macrophages. RAW264.7 cells were treated with Medrysone in the basal or IL-4 induced model for 24 h, and the expression of CD206, which represents the M2-like macrophages was detected by flow cytometry assay. As shown in (Fig. 1A), Medrysone increased the percentage of CD206-positive cells in basal and IL-4 treated conditions. Similar results were also observed in BMDMs (Bone Marrow-Derived Macrophages) (shown in (Fig. 1B)). Meanwhile, we analyzed the M2-like-associated genes by quantitative real-time PCR assay. As shown in (Fig. 1C), Medrysone up-regulated the mRNA expression level of M2 maker genes, including, *Mrc1*, *IL10*, *Fizz1, Arg1* and *CD11b* in RAW264.7 cells. These data suggested that Medrysone promoted M2 polarization of macrophages in vitro.

### Medrysone promoted VEGF and CCL2 secretion in IL-4 induced M2-like polarization

**Fig. 2:**
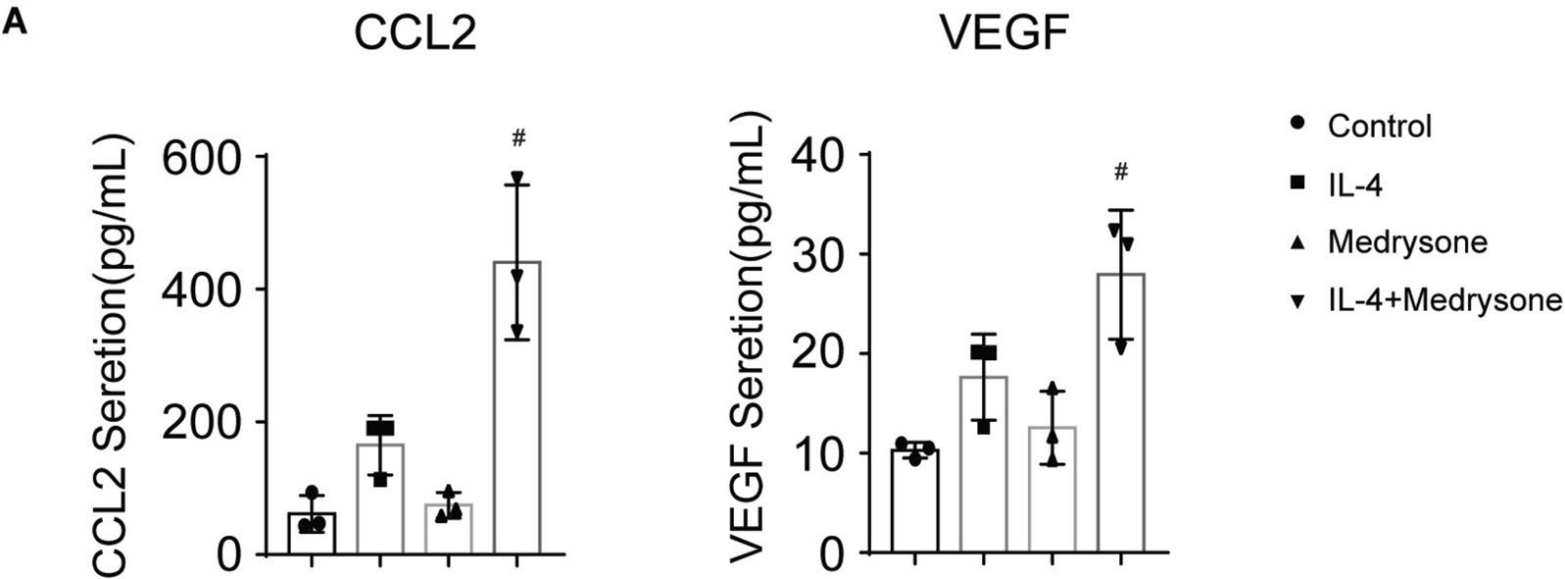
Medrysone promoted VEGF and CCL2 secretion. **A** The secretion of CCL2 and VEGF in Raw264.7 were detected by Elisa assay after the treatment of Medrysone (5 μM) and IL-4 (10 ng/mL) for 24 h. The data are presented as the mean ± SD of triplicate experiments. Statistical significance was determined by one-way ANOVA with Dunnett’s post hoc test. *#, p* < 0.05

During inflammatory damage repair, VEGF and CCL2 secreted by M2-like macrophages could promote tissue damage repair [14]. Therefore, we investigated whether Medrysone could promote VEGF or CCL2 secretion in the presence of IL-4. By enzyme-linked immunosorbent assay (ELISA), we found that both VEGF and CCL2 secretion were significantly increased in the combination group of Medrysone and IL-4 (shown in (Fig. 2A)). This result suggested that Medrysone may participate in the repair of cornea in inflammation by secreting VEGF and CCL2.

### p-STAT6 was involved in Medrysone promoted M2-like polarization

**Fig. 3:**
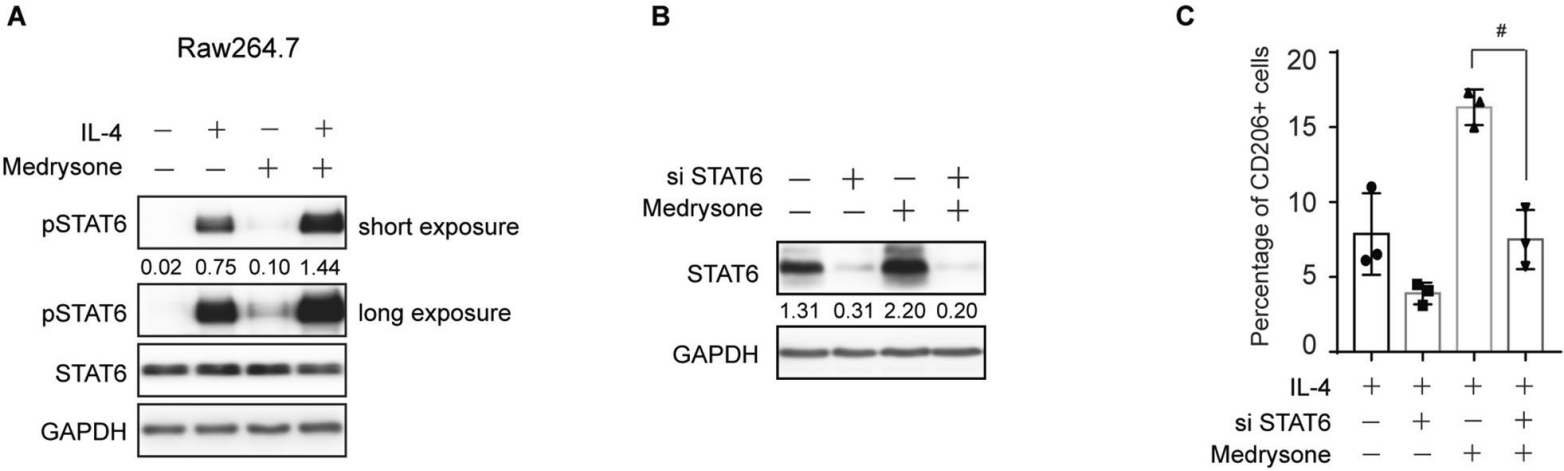
Medrysone induced M2-like polarization by IL4-STAT6 pathway. **A** Medrysone increased the phosphorylation of STAT6. Raw264.7 was treated with or without Medrysone (5 μM) and IL-4 (10 ng/mL) for 24 h, and the expression of p-STAT6 was detected by Western Blotting. The images were quantified using Image J grayscale analysis and represented as relative pSTAT6 to STAT6. **B** and **C** The up-regulation of CD206 expression induced by Medrysone was abolished by the absence of STAT6. Raw264.7 was treated with Medysone (5 μM) for 24 h after transfection of NC or siSTAT6, and the expression of CD206 was detected by flow cytometry. The images were quantified using Image J grayscale analysis and represented as relative STAT6 to GAPDH. The data are presented as the mean ± SD of triplicate experiments. Statistical significance was determined by one-way ANOVA with Dunnett’s post hoc test. *#, p* < 0.05

Previous studies have shown that IL-4 induces M2-like polarization by activating the p-STAT6 pathway [15]. Therefore, we further explored whether IL-4/p-STAT6 pathway was involved in Medrysone-promoted M2-like polarization. As shown in (Fig. 3A), Medrysone significantly enhanced the phosphorylation of STAT6 in RAW264.7 cells. Furthermore, the M2-like polarization induced by Medrysone was abolished in the absence of STAT6 (shown in (Fig. 3B, C)). Taken together, these results indicated that Medrysone could induce M2 polarization by activating p-STAT6 pathway.

### Medrysone enhanced migration-promoting feature of M2-like macrophages

**Fig. 4:**
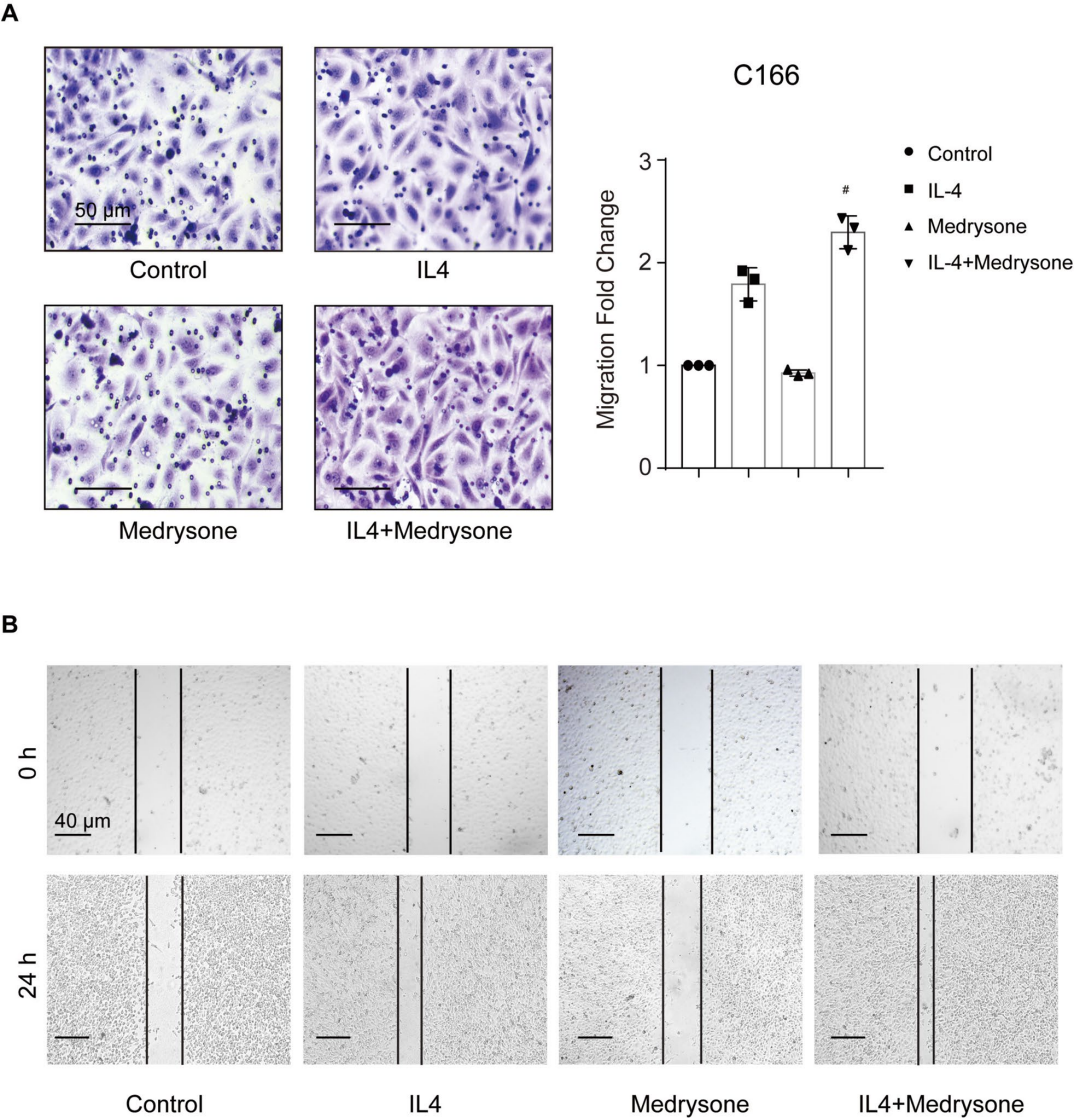
Medrysone promoted vascular endothelial cell migration. **A** Medrysone enhanced the migration of C166 cells. RAW264.7 cells were treated with Medrysone (5 μM) and IL-4 (10 ng/mL) for 24 h, and the supernatant medium was collected as macrophages-conditioned medium (CM). C166 cells were seeded into trans-well chamber and cultured in CM condition for 24 h. Scale bar: 50 μm. **B** C166 cells in 24-well plates were incubated with CM after scratching. Scale bar: 40 μm. The data are presented as the mean ± SD of triplicate experiments. Statistical significance was determined by one-way ANOVA with Dunnett’s post hoc test. *#, p* < 0.05

Multiple evidence has proved that M2-like polarization tumor-associated macrophages could promote cell migration and invasion, so we investigated the effects of Medrysone on vascular endothelial cell migration. Conditional medium was obtained when the macrophages were treated with Medrysone for 24 h. We then evaluated the migrating ability of murine vascular endothelials in conditional medium by using wound-healing assay and migration assay. As shown in (Fig. 4A), Medrysone significantly enhanced the migration of murine vascular endothelial C166 cells, and similar result was obtained from wound healing assay (shown in (Fig. 4B)).

### Medrysone promoted corneal injury repair by inducing M2 polarization of macrophages in vivo

**Fig. 5:**
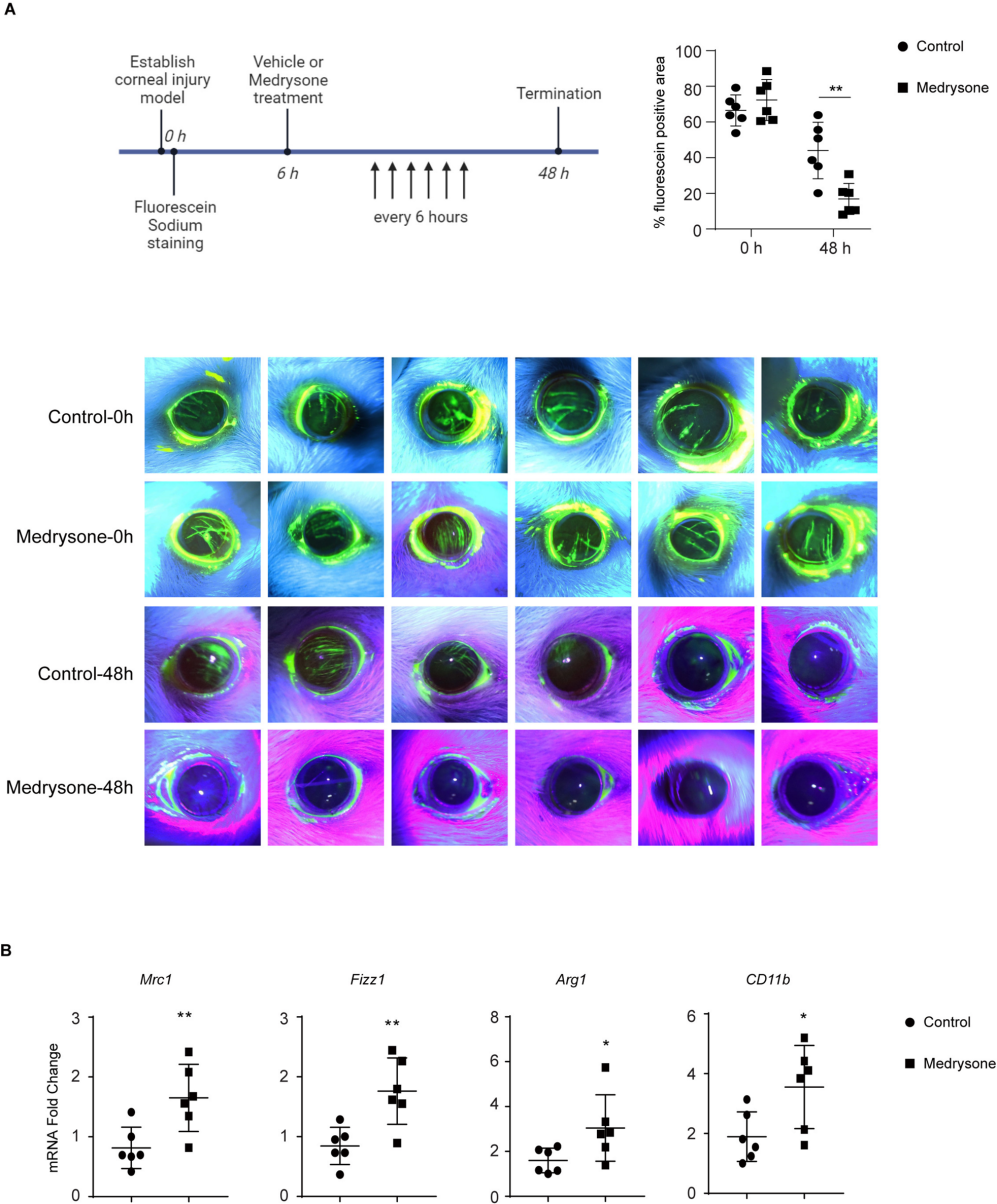
Medrysone promoted corneal injury repair in vivo. The cornea of the rats was mechanically damaged with ophthalmic surgical instruments. After the operation, the rats were randomly divided into the control group and Medrysone group. The Medrysone group received Medrysone treatment every 6 h, and the cornel injury damage was detected by fluorescein sodium staining and recorded by slit lamp (*n* = 6). **A** The timeline and results of fluorescein sodium staining of the cornea. **B** The mRNA expression of macrophage M2-related genes in corneal tissue was detected by quantitative real-time PCR assay. Statistical significance was determined by Students’ t test. *, p < 0.05, **, p < 0.01

To explore the effect of Medrysone on the corneal injury repair process, rat corneal mechanical injury model was established. We generated a rat corneal mechanical injury model by using ophthalmic surgical instruments, the carving groove which represents the degree of corneal injury was observed by fluorescein sodium staining. After the operation, the rats were randomly divided into 2 groups. The Medrysone treated group was given Medrysone every 6 h, and after 48 h, the corneal damage repair was investigated by fluorescein sodium staining again. As shown in (Fig. 5A), the treatment of Medrysone significantly promoted the corneal damage repair process. Furthermore, we investigated whether Medrysone could induce M2-like polarization in vivo, by using quantitative real-time PCR assay, we confirmed that Medrysone induced the mRNA expression level of M2 marker genes, including *Mrc1, Fizz1, Arg1* and *CD11b* (shown in (Fig. 5B)).

## Discussion

Corneal is the outermost transparent part of the eyes, directly exposed to the environment and thus highly susceptible to chemical burns, mechanical damage, and infection [16, 17]. Incomplete repair and excessive healing of the cornea can lead to serious eye complications and cause different levels of vision defects.

Inflammation was shown to play an important role in the corneal injury healing process [18]. The complex inflammatory environment in the corneal illness affects injured corneal repair process, which is composed of various inflammatory cells and pro-inflammatory or anti-inflammatory factors. Current research suggests that a variety of growth factors, such as EGFR (Epidermal Growth Factor Receptor), FGF (Fibroblast Growth Factor), PDGF (Platelet-Derived Growth Factor) and IGF (Insulin Growth Factor) play a key role in corneal healing, and the inflammatory factors such as TNF-α (Tumor Necrosis Factor-α) and IL-6 (Interleukin-6) are also involved in the process of corneal injury repair [19, 20]. However, excessive activation of inflammation often has adverse effects on injury repair process and may cause perforation or fibrosis of the cornea. Therefore, taking the inflammatory response into control may affect the outcome of corneal injury.

Pharmacologically controlling unwanted effects of wound healing process may avoid corneal perforation or scar formation. Anti-TGF-β (Transforming Growth Factor-β) treatments such as rosiglitazone, ROCK (Rho-associated kinase) inhibitor or trichostatin A were shown to be of clinical benefit in pharmacological management of wound healing [21–23]. Our study found that Medrysone promoted corneal injury repair by inducing macrophage M2 polarization and regulating inflammatory response, which may provide a new strategy for clinical ophthalmic treatment.

The application of corticosteroids in corneal injury has always been controversial [24–26]. Several studies suggested that the use of corticosteroids in the early stage of injury will accelerate the process of corneal dissolution and affect the repair of the injury [27]. However, some studies revealed that the corticosteroids don’t have any adverse effect on corneal repair, and due to their anti-inflammatory effect, corticosteroids could reduce scarring significantly [28]. In our study, we found that the corticosteroid drug Medrysone significantly promoted corneal injury repair, which was related to its promotion of M2-like macrophages and secretion of VEGF and CCL2. However, Medrysone can induce VEGF secretion, which is a strong promoter of corneal neovascularization in clinic. In our experiment, we did not observe corneal neovascularization in Medrysone short term treatment, whether long administration of Medrysone will lead to neovascularization need further study.

## Conclusion

Taken together, our study showed that Medrysone could significantly induce M2- polarization of macrophages and promote corneal injury repair.

### Supplementary Information

**Additional file 1.** 

** Additional file 2.**

## Data Availability

Data generated for the current study are available from the corresponding author on reasonable request.
