# Supplementary material for: Medrysone promotes corneal injury repair by promoting M2-like polarization of macrophages
Source: BMC Ophthalmol. 2023 Dec 11;23:503. doi: 10.1186/s12886-023-03234-3 (PMC10712160; doi:10.1186/s12886-023-03234-3)
Supplement: Supplementary file 1 — Additional file 1. [file 12886_2023_3234_MOESM1_ESM.docx]

| Table S1. Actual numbers for Figure 1C | | |
| --- | --- | --- |
| Group | | mean ± SD |
| *Mrc1* | Control | 1 |
|  | IL-4 | 7.30 ± 2.23 |
|  | Medrysone | 1.73 ± 0.42 |
|  | IL-4+Medrysone | 14.60 ± 3.46 |
| *IL10* | Control | 1 |
|  | IL-4 | 7.51 ± 4.87 |
|  | Medrysone | 1.68 ± 0.46 |
|  | IL-4+Medrysone | 15.63 ± 7.73 |
| *Fizz1* | Control | 1 |
|  | IL-4 | 7.95 ± 1.95 |
|  | Medrysone | 1.89 ± 0.53 |
|  | IL-4+Medrysone | 16.36 ± 6.16 |
| *Arg1* | Control | 1 |
|  | IL-4 | 7.33 ± 2.22 |
|  | Medrysone | 1.75 ± 0.65 |
|  | IL-4+Medrysone | 17.18 ± 6.56 |
| *CD11b* | Control | 1 |
|  | IL-4 | 3.04 ± 0.74 |
|  | Medrysone | 2.22 ± 0.77 |
|  | IL-4+Medrysone | 7.38 ± 2.50 |

| Table S2. Actual numbers for Figure 2 | | |
| --- | --- | --- |
| Group | | mean ± SD |
| CCL2 | Control | 61.52 ± 27.81 |
|  | IL-4 | 165.02 ± 44.75 |
|  | Medrysone | 74.56 ± 19.43 |
|  | IL-4+Medrysone | 440.60 ± 116.74 |
| VEGF | Control | 10.30 ± 0.79 |
|  | IL-4 | 17.62 ± 4.31 |
|  | Medrysone | 12.55 ± 3.68 |
|  | IL-4+Medrysone | 27.94 ± 6.48 |

| Table S3. Actual numbers for Figure 3C | |
| --- | --- |
| Group | mean ± SD |
| NC | 7.87 ± 2.72 |
| siSTAT6 | 3.90 ± 0.72 |
| Medrysone | 16.33 ± 1.19 |
| siSTAT6+Medrysone | 7.50 ± 1.97 |

| Table S4. Actual numbers for Figure 4A | |
| --- | --- |
| Group | mean ± SD |
| Control | 1 |
| IL-4 | 1.79 ± 0.16 |
| Medrysone | 0.93 ± 0.03 |
| IL-4+Medrysone | 2.14 ± 0.19 |

| Table S5. Actual numbers for Figure 5B | | |
| --- | --- | --- |
| Group | | mean ± SD |
| *Mrc1* | Control | 0.81 ± 0.35 |
|  | Medrysone | 1.65 ± 0.56 |
| *Fizz1* | Control | 0.84 ± 0.31 |
|  | Medrysone | 1.76 ± 0.55 |
| *Arg1* | Control | 1.60 ± 0.55 |
|  | Medrysone | 3.04 ± 1.48 |
| *CD11b* | Control | 1.89 ± 0.83 |
|  | Medrysone | 3.55 ± 1.39 |
