# Supplementary figures and images for: Medrysone promotes corneal injury repair by promoting M2-like polarization of macrophages
Source: BMC Ophthalmol. 2023 Dec 11;23:503. doi: 10.1186/s12886-023-03234-3 (PMC10712160; doi:10.1186/s12886-023-03234-3)

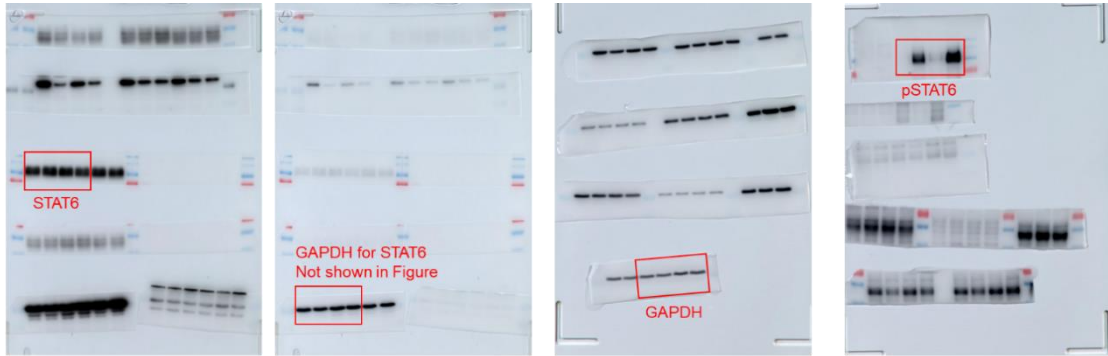

Raw data for Figure 3A

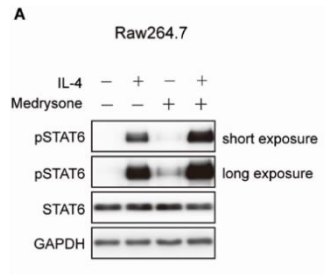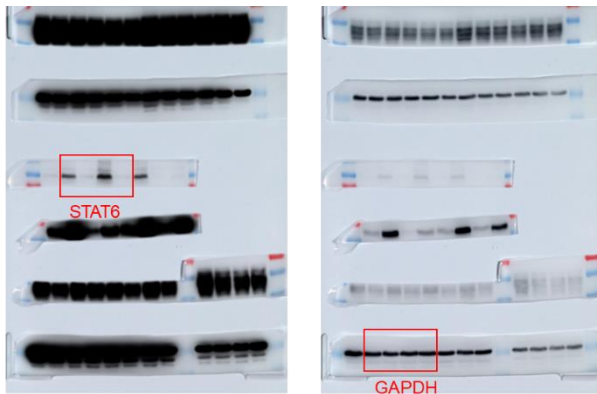

Raw data for Figure 3B

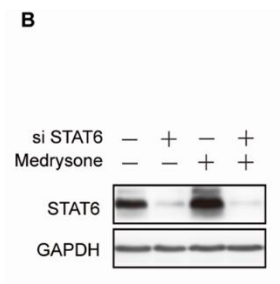

Supplement: Supplementary file 2 — Additional file 2. [file 12886_2023_3234_MOESM2_ESM.pdf]
